# Task-dependent increases and decreases of BOLD signal in theory of mind brain regions during strategic social interaction

**DOI:** 10.3389/fncir.2026.1741762

**Published:** 2026-04-08

**Authors:** Maya Zheltyakova, Maxim Kireev, Irina Knyazeva, Artem Myznikov, Vladimir Kiselev, Mikhail Didur, Denis Cherednichenko, Alexander Korotkov

**Affiliations:** 1 N.P. Bechtereva Institute of the Human Brain, Russian Academy of Science, Saint Petersburg, Russia; 2 Institute for Cognitive Studies, Saint Petersburg State University, Saint Petersburg, Russia

**Keywords:** affective ToM, cognitive ToM, functional MRI, mentalizing, rock-paper-scissors, strategic game, task-modulated functional connectivity

## Abstract

Theory of Mind (ToM) is known as the capacity to infer others’ thoughts, intentions, and emotions, supported by a distributed neural brain network, including the medial prefrontal cortex (mPFC), temporoparietal junction (TPJ), inferior frontal gyrus (IFG), and precuneus. Although the Rock-Paper-Scissors (RPS) game is used to study the cognitive ToM domain, previous fMRI studies had methodological limitations, including lack of appropriate control conditions and the absence of analyses addressing the directionality of BOLD signal changes. The present fMRI study employed a modified RPS paradigm designed to overcome these limitations. Forty-six healthy adults performed the RPS game and a control task. Whole-brain analyses contrasted neural activity and task-modulated functional connectivity (TMFC) between these conditions and examined BOLD signal changes relative to baseline. In contrast to prior findings of BOLD signal suppression below baseline in affective ToM tasks, RPS elicited increased BOLD responses in canonical ToM regions, including the mPFC, bilateral TPJ, IFG, and precuneus, as well as additional frontal, cingulate and visual regions. TMFC analyses converged with these findings, demonstrating increased RPS-related functional interactions between the bilateral TPJ and precuneus with the left IFG, and between the mPFC and the right TPJ with the right IFG. Additionally, greater deactivation (negative BOLD deflection) below baseline during RPS was observed in the midcingulate cortex and opercular regions bilaterally. These findings extend current understanding of ToM network functioning by demonstrating that the engagement of its affective and cognitive domains manifest through TMFC changes and directionally distinct neural responses.

## 1 Introduction

The term Theory of Mind (ToM) refers to the ability to represent the mental states of others and to infer their goals, intentions, beliefs, and emotions (Premack and Woodruff, 1978). The process of applying this ability is commonly termed as mentalizing. The neural substrates of ToM have been widely studied, revealing a distributed network that includes the bilateral temporoparietal junction (TPJ), inferior frontal gyrus (IFG), lateral temporal cortex, medial prefrontal cortex (mPFC), and precuneus (Van Overwalle, 2009; Mar, 2011; Schurz et al., 2014, 2021; Molenberghs et al., 2016; Arioli et al., 2021; Diveica et al., 2021; Balgova et al., 2024).

Recent efforts to study mentalizing under more ecological and interactive conditions have led to the use of socially engaging experimental paradigms, such as the Rock-Paper-Scissors (RPS) game. Importantly, RPS has been shown to induce a specific component of online mentalizing, namely the adoption of an intentional stance, requiring participants to infer an opponent’s beliefs, desires, and intentions to guide their actions and maximize performance (Gallagher et al., 2002; Chaminade et al., 2012). This fairly well-known game, serving as an intuitive form of social interaction, has been adapted for multiple neuroimaging modalities, including EEG (Perry et al., 2011; Wang et al., 2020; He et al., 2024), MEG (Bu et al., 2023), fMRI (Paulus et al., 2004), and functional near-infrared spectroscopy (Kikuchi et al., 2007; Yamauchi et al., 2013; Kayhan et al., 2022; Zhang et al., 2024). The first neuroimaging PET study using RPS demonstrated activation of the mPFC (Gallagher et al., 2002), laying the foundation for subsequent fMRI research in this field.

However, most fMRI studies using RPS do not provide clear evidence about how the ToM system operates during real social interaction. In several experiments, participants only observed others playing the game (Plata Bello et al., 2015; Shimada et al., 2016). In others, they lacked freedom of choice, since participants were told which gesture the opponent would choose and were instructed either to win or lose (Matsubara et al., 2004; Kadota et al., 2010) or to intentionally lose, which mainly engaged inhibitory control over stereotypical responses (Kadota et al., 2009). The free choice is an important condition for ToM engagement, which is supported by a meta-analysis of studies on intentional deception, showing that voluntary lying (unlike instructed lying) increases activation in the dorsal anterior cingulate cortex, right TPJ, and bilateral temporal poles (Lisofsky et al., 2014).

In addition, many studies had a focus on other aspects of the RPS task. Some compared motor execution with feedback or gesture observation (Paulus et al., 2005; Dinstein et al., 2007, 2008), while others investigated neural mechanisms of feedback processing (Sugimoto et al., 2021) or examined individuals with substance dependence and various personality traits (Stewart et al., 2013, 2014a, 2014b, 2017; Abu-Akel et al., 2017). Thus, in general RPS-based neuroimaging research did not create conditions optimal for investigating the neural basis of mentalizing.

Despite these limitations, several studies reported activation in ToM-related regions during RPS task such as the mPFC, right TPJ, and precuneus (Chaminade et al., 2012; Abu-Akel et al., 2020). However, these studies lacked proper control conditions, since comparisons were made between different types of opponents, such as human, artificial intelligence, or robot (Chaminade et al., 2012; Abu-Akel et al., 2017, 2020), or based on whether the opponent’s behavior was intentional or algorithmic (Abu-Akel et al., 2017, 2020). Furthermore, the study by Chaminade et al. (2012) used too few trials per condition (21 per opponent), which limited the statistical power according to current standards and constricts further investigation of ToM network activity with the usage of such data for functional interactions analysis (Turner and Miller, 2013; Cisler et al., 2014; Masharipov et al., 2024).

Another major limitation is that, except for one study (Chaminade et al., 2012), most RPS neuroimaging reports did not analyze BOLD signal changes relative to baseline. This omission restricts interpretation of the functional involvement of ToM regions. Previous studies using other social tasks showed that these same regions often display BOLD signal decreases relative to baseline (also known as deactivation). For a long time, such below-baseline deactivations were regarded as artifacts. For example, Mitchell et al. (2002) found minimal deactivation in the mPFC, superior temporal cortex, and intraparietal sulcus during person-judgment tasks, whereas object-related tasks produced stronger deactivation. Similarly, classical ToM tasks such as reading stories about false beliefs (vs. physical stories) produced higher responses in the right TPJ (Mitchell, 2008), mPFC (Dodell-Feder et al., 2011), and precuneus (Saxe and Kanwisher, 2003), though all conditions showed BOLD signal decreases below baseline. In addition, Zheltyakova et al. (2025) demonstrated greater deactivation in the mPFC, angular and supramarginal subregions of the TPJ, and precuneus during a task requiring identification of affective mental states from the gaze expressions compared with an age-identification control condition. However, both increased activation and deactivation below baseline may indicate functional engagement. The evidence is provided by comparing functional activity and connectivity in ToM network brain regions during tasks requiring mentalizing, e.g., simple deception and its sophisticated form, conducted by telling the truth with the expectation that an opponent will not believe it (Zheltyakova et al., 2020, 2022). During sophisticated deception the left TPJ had a below-baseline BOLD signal but increased compared to simple deception functional interactions with the right TPJ—brain area with increased above-baseline BOLD signal. Increased functional coupling suggests that, despite deactivation below baseline, brain regions may participate in the task-related brain network.

This creates an apparent contradiction. On the one hand, several studies report increased activation in ToM regions (mPFC, right TPJ, and precuneus) during RPS. On the other hand, evidence suggests that BOLD signals in these regions may decrease below baseline in other social tasks. Resolving this inconsistency requires a detailed analysis of the directionality of BOLD signal changes during RPS.

The present study aimed to clarify the characteristics of ToM system engagement during social interaction using a modified fMRI version of the RPS game. This modification addressed the methodological limitations of previous RPS-based research by allowing free choices, including a control condition requiring selection among three geometric figures, and by increasing the number of trials per condition.

## 2 Methods

### 2.1 Participants

In this fMRI study using the RPS game, 48 right-handed healthy volunteers (20 males, 28 females; mean age = 23 ± 4.3 years) were scanned. None of the participants reported any history of neurological or psychiatric disorders. Handedness was assessed using the Edinburgh Handedness Inventory (Oldfield, 1971). All participants provided written informed consent prior to participation. The study procedures were approved by the Ethics Committee of the N.P. Bechtereva Institute of the Human Brain, Russian Academy of Sciences.

### 2.2 Stimuli and procedure

To examine the involvement of the ToM neural system in social interactions, the classical version of the RPS game played against an opponent was used as the primary experimental paradigm (for detailed description of the task see Supplementary Data 1.1). Before the task, participants were instructed that they would play RPS against a real opponent (one of the experimenters) and were introduced to each other. In reality, all opponent responses were preprogrammed to ensure an equal number of successful, unsuccessful, and neutral outcomes for each participant, thereby controlling for opponent-related and learning effects and enabling appropriate intersubject comparisons.

**FIGURE 1:**
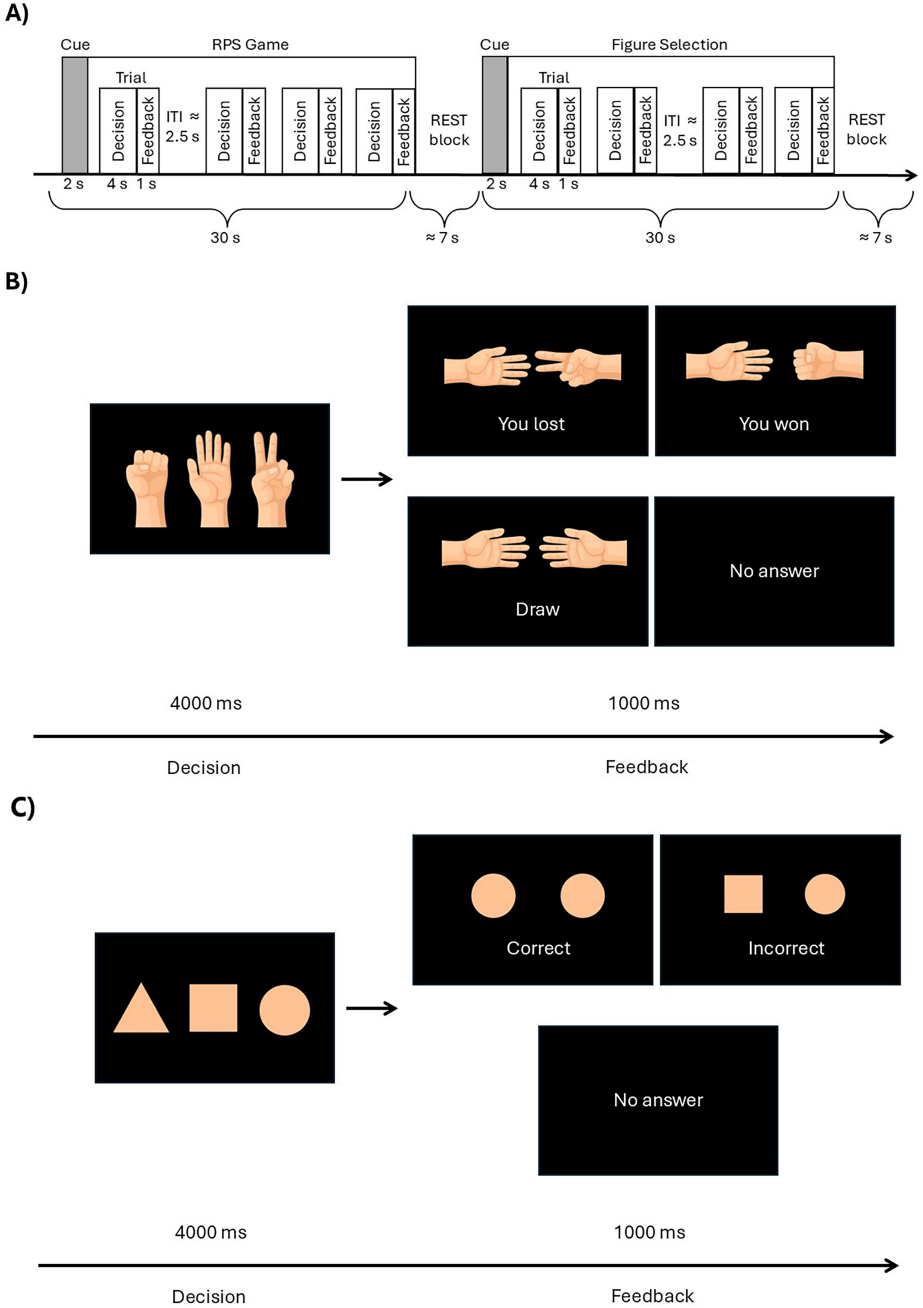
Schematic representation of the experimental procedure and experimental trials under the social and control conditions. **(A)** Experimental procedure. **(B)** Rock-paper-scissors (RPS) game trial, illustrating the decision phase and all feedback types. Participants are instructed to choose the gesture that they think will allow them to win against the opponent by pressing the button corresponding to the left (rock), center (paper) or right (scissors) option. During the feedback phase, the participant’s choice is shown on the left side of the display, and the opponent’s choice on the right. **(C)** Figure-selection (control) trial, also depicting the decision phase and all possible feedback types. Participants are verbally instructed, which shape is the correct one prior to the beginning of the session, and select its position by pressing the button corresponding to the left, center or right option. In the illustration, the correct shape is circle and, thus, participants have to select the option on the right to answer correctly. During the feedback phase, the participant’s choice is presented on the left, while the target figure (a circle in this example) is shown on the right.

In each RPS game trial, participants freely selected one of three gestures (rock, paper, or scissors) using corresponding button presses (see Supplementary Data 1.2) to win against the opponent and saw the game feedback after each trial (see Figure 1B). The control condition required participants to select the correct figure from three geometric shapes (triangle, square, or circle) presented on the screen and were provided with the feedback (see Figure 1C).

Each of the three experimental sessions lasted approximately 17 min (see Figure 1A). Sessions included alternating task and rest blocks, with a fixation cross presented during rest periods. Each session comprised 24 task blocks and 24 rest blocks. Rest blocks were pseudo-randomly jittered from 2 to 12 s in 2-s increments according to pre-defined weights (mean ≈ 7 s). Task blocks included 12 RPS game blocks and 12 control figure selection blocks presented in random order.

At the beginning of each task block, a cue indicating the condition type (“game” or “figures”) appeared on the screen for 2 s. In the RPS condition, the opponent’s photograph was also displayed alongside the word “game.” The interval between the start of scanning and the first cue was 5.5 s. The fixation interval between the preparatory cue and the start of the block was pseudo-randomly jittered from 1.5 to 3.5 s. in 0.5-s increments (mean ≈ 2 s).

Each block contained four trials. A single trial lasted 5 s: participants had 4 s to make a decision (choose a gesture or figure presented as schematic icons), followed by 1 s of feedback. The inter-trial interval was pseudo-randomly jittered between 1 and 4 s in 1-s increments (mean ≈ 2.5 s). The total duration of each block was 30 s. Schematic representations of the RPS game and figure selection control conditions are shown in Figure 1.

### 2.3 fMRI data acquisition and preprocessing

The study was conducted using two 3.0 Tesla MRI scanners: Philips Achieva 3.0T (30 participants) and GE SIGNA Architect 3.0T (18 participants).

#### 2.3.1 Philips achieva 3.0T

Structural images were acquired using a T1-weighted 3D fast field echo (T1W–3D–FFE) sequence with the following parameters: repetition time (TR) = 2.5 ms, echo time (TE) = 3.1 ms, and flip angle = 30°. A total of 130 axial slices were obtained (slice thickness = 0.94 mm; in-plane resolution = 1 × 1 mm; field of view (FOV) = 240 × 240 mm; acquisition matrix = 256 × 256).

Functional T2*-weighted images were acquired using a single-shot echo-planar imaging (EPI) sequence (TE = 35 ms; flip angle = 90°; FOV = 208 × 208 mm; matrix = 128 × 128). The sequence continuously collected 32 axial slices (slice thickness = 3.5 mm; voxel size = 3 × 3 × 3.5 mm), covering the entire cerebral cortex and most of the cerebellum, oriented parallel to the structural scan. The TR for one functional volume was 2,000 ms.

#### 2.3.2 GE SIGNA architect 3.0T

Structural images were acquired using a T1-weighted MPRAGE sequence (TR = 2,605 ms; TE = 2.4 ms; inversion time (TI) = 1,000 ms; flip angle = 8°). A total of 180 axial slices were obtained (slice thickness = 0.9 mm; in-plane resolution = 0.9 × 0.9 mm; FOV = 240 × 240 mm; matrix = 256 × 256).

Functional T2*-weighted images were obtained using a single-shot EPI sequence (TE = 21.8 ms; flip angle = 52°; FOV = 208 × 208 mm; matrix = 86 × 86). Each volume consisted of 48 axial slices (slice thickness = 2.8 mm; voxel size = 2.4 × 2.4 × 2.8 mm), covering the entire cortex and most of the cerebellum. Functional images were aligned with the corresponding structural scans. The TR was 960 ms.

To minimize head motion during scanning, participants’ heads were stabilized using an MRI-compatible fixation collar (Shantz brace).

### 2.4 fMRI data preprocessing

Preprocessing included the following steps: spatial realignment of functional images, slice-time correction, co-registration of structural and functional images, segmentation of structural images into tissue classes, normalization to the Montreal Neurological Institute (MNI) standard space, and spatial smoothing using an 8 mm FWHM Gaussian kernel. All preprocessing and statistical analyses were performed using SPM12 (Statistical Parametric Mapping; Wellcome Department of Imaging Neuroscience, London, United Kingdom)^1^ implemented in MATLAB (MathWorks Inc., Natick, MA, United States).

### 2.5 Statistical analysis of fMRI data

To assess the BOLD signal changes at the first (individual subject) level, a general linear model (GLM) was applied to estimate event-related effects. The model included two decision-locked regressors representing two task conditions (events): (1) the RPS game (social interaction condition) and (2) the figure selection task (control condition). Event duration was equal to response time (see section 3.1). Group average number of trials was 141.5 (± 2.4 *SD*) and 142.5 (± 1.9 *SD*) for the control and social interaction condition, respectively. To regress out effects of no interest the GLM also included one feedback-locked regressor with event duration equal to 1 s, corresponding to the feedback presentation phase irrespective of the type of task condition, as well as one nuisance regressor modeling trials without response. Twenty-four motion parameters were included as nuisance regressors (Friston et al., 1996). All decision-locked regressors were created by convolving the canonical hemodynamic response function (HRF) with temporal characteristics of decision phase (onset times at the beginning of the trial).

**FIGURE 2:**
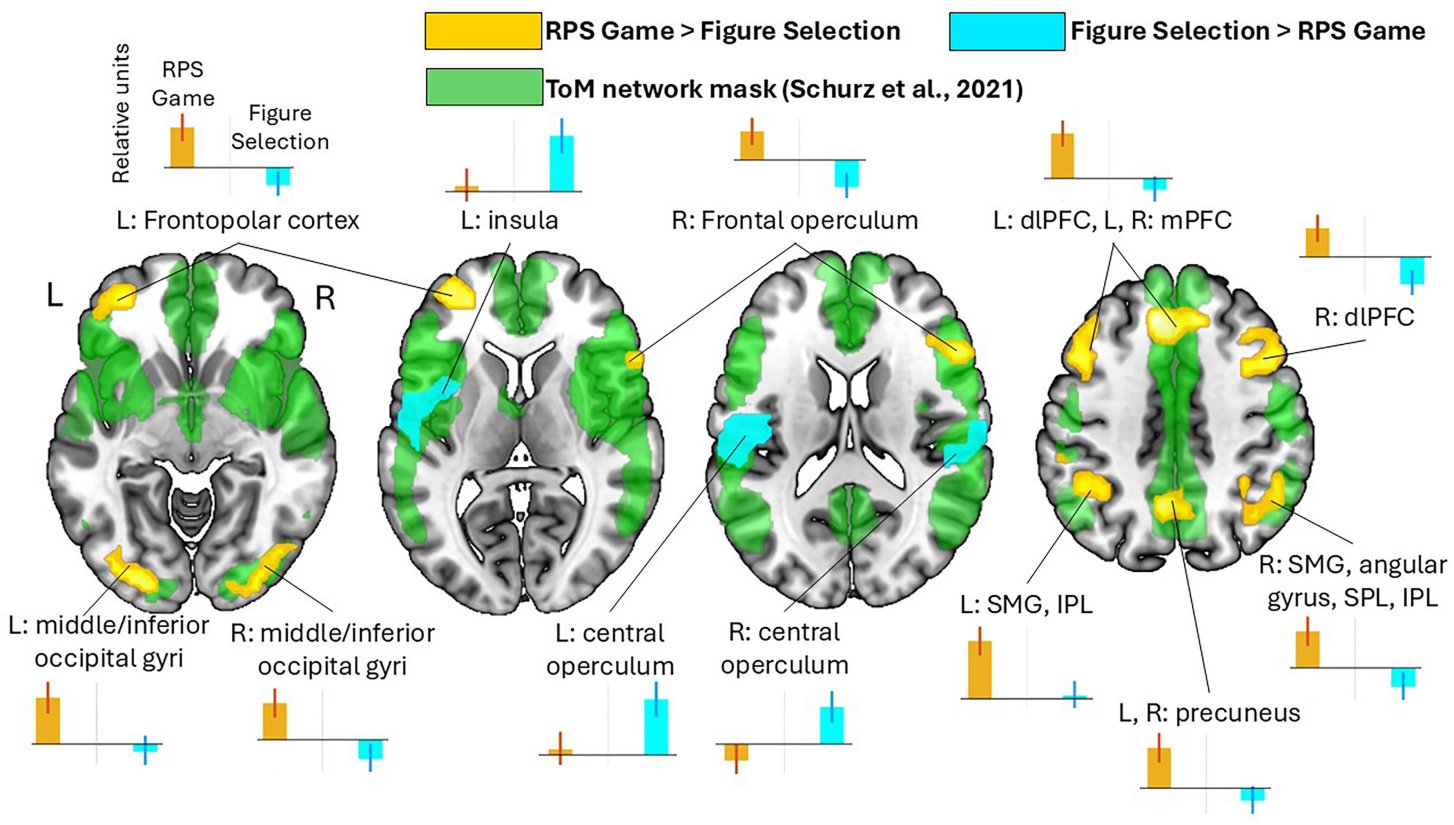
Clusters of increased (in orange) and decreased (in teal) BOLD signals associated with performance in the socially interactive RPS game (*p* < 0.001 uncorrected at the voxel level, followed by cluster-level FWE correction at *p* < 0.05). The ToM network clusters are presented in green, based on the ToM network masks from the meta-analysis by Schurz et al. (2021) (https://neurovault.org/collections/9936/, accessed September 3, 2024). Plots show effect sizes for conditions of interest (vs. baseline) with 90% confidence intervals.

At the first level, comparisons of interest were created for decision-locked regressors and included “RPS Game > Figure Selection” betas contrasts for assessing the effect of playing the game. “Condition > Rest” contrasts were used to identify BOLD signal changes for clusters revealed in the “RPS Game” vs. “Figure Selection” comparison. At the second (group) level, a one-sample *t*-test was performed on the “RPS Game > Figure Selection” betas contrasts to identify brain regions associated with the ToM-related social condition. To analyze the effect of the scanner on the level of BOLD signal we included corresponding covariates to the one-sample model. Additionally, to assess the directionality of significant BOLD signal changes and create plots of effect sizes for conditions of interest (see Figure 2) full-factorial model for betas revealed in “RPS Game > Rest” and “Figure Selection > Rest” comparisons was estimated.

To assess the task-modulated functional connectivity (TMFC) we performed the beta-series correlation using least-squares separate approach (BSC-LSS; Mumford et al., 2012), as it is implemented in the TMFC toolbox^2^ for ROIs (regions of interest; see Supplementary Figure 9) selected on the basis of results of BOLD signal analysis and the Brainnettom anatomical brain atlas (Fan et al., 2016; for details see Supplementary Data 1.3).

Statistical inference for activation and functional connectivity analyses was performed using classical frequentist statistics with a significance threshold of *p* < 0.001 uncorrected at the voxel level, followed by cluster-level FWE correction at *p* < 0.05. A gray matter mask derived from individual T1 segmentation maps was applied to restrict analyses to brain tissue.

## 3 Results

### 3.1 Behavioral results

Two participants were excluded from the analysis due to an excessive number of missed trials (26 and 32, respectively). The final sample thus included 46 participants (19 males, 27 females; mean age = 22.9 ± 4.2 years). The number of wins, losses, and draws in the RPS condition was predefined, the same for all subjects, and equal (33% out of all trials). The success rate in the control condition was approximately 100% and did not differ across participants. Response time analysis revealed no significant difference between the RPS game and the figure-selection control condition (*p* = 0.065). Mean response time was 947 ± 290 ms for the RPS condition and 880 ± 187 ms for the control condition.

To provide behavioral evidence for hypothesized involvement of mentalizing process, three behavioral signatures were calculated, indicating opponent modeling during RPS game (for detailed analyses and results description see Supplementary Data 1.4.2). First, the probability of repeating the same gesture on consecutive trials (*M* = 0.274, *SD* = 0.087) was significantly below the random baseline of 1/3 [*t*(45) = -4.63, *p* < 0.001, *d* = -0.68], indicating active avoidance of predictable patterns in order to outwit the opponent (Dyson, 2019; Brockbank and Vul, 2021, 2024). Second, the win-stay rate (0.258) was significantly below 1/3 [*t*(45) = -4.67, *p* < 0.001, *d* = -0.69]. This is opposite to the reinforcement learning (RL) prediction and below random chance. Participants avoided repeating a winning gesture, which is consistent with the belief that the opponent would detect and counter a successful strategy (Sundvall and Dyson, 2022). Critically, responses in general were largely outcome-independent: stay rates were similar after wins (0.258), losses (0.293), and draws (0.264), in contrast to the strong outcome dependence predicted by RL models. Third, when participants switched gestures on two consecutive trials, they preferentially cycled forward to the remaining unused gesture [probability of returning to the gesture from two trials back: 0.444 vs. 0.500 expected under RL; *t*(45) = –4.23, *p* < 0.001, *d* = -0.62]. Such results revealed a second-order sequential dependency, which requires modeling how one’s own actions shape the opponent’s expectations and first-order RL models cannot produce (Hampton et al., 2008; Crawford et al., 2013; Wang et al., 2014; Dyson, 2019).

### 3.2 fMRI results

Performance in the RPS game versus the figure selection (control) condition was associated with the increased BOLD signal in several brain regions, including the left frontopolar cortex (FPC), right frontal operculum, bilateral dorsolateral (including the inferior, middle, and superior frontal gyri and precentral gyrus) and dorsomedial PFC, anterior and middle cingulate cortex, supplementary motor area, bilateral supramarginal gyrus (SMG), inferior parietal lobule, and precuneus, right angular gyrus (AG) and superior parietal lobule (SPL), as well as bilateral occipital cortex (see Figure 2 and Table 1 for statistical details).

**TABLE 1:** Clusters of increased and decreased BOLD signals associated with performance in the socially interactive RPS game (*p* < 0.001 uncorrected at the voxel level, followed by cluster-level FWE correction at *p* < 0.05).

No.	Anatomical localization	Cluster size	*T*-value	Peak coordinates in the standard anatomical MNI space		
				*x*	*y*	*z*
RPS game > Figure selection						
1	L: inferior/middle/superior frontal gyri, precentral gyrus L, R: dmPFC, SMA, anterior/middle cingulate cortex	862	5,013	–9	32	44
			4,949	9	29	50
			4,497	–3	20	50
2	L: middle/inferior occipital gyri	131	4,436	–21	–91	–10
			4,180	–33	–85	–10
3	R: middle/inferior occipital gyri	215	4,392	48	–76	–1
			4,108	21	–97	2
			4,022	42	–76	–13
4	L: frontopolar cortex	219	4,358	–33	47	–4
			4,322	–27	50	2
5	L, R: precuneus	100	4,329	–6	–58	44
6	R: inferior/middle/superior frontal gyri, frontal operculum, precentral gyrus	484	4,302	36	8	47
			4,299	45	17	50
			4,256	54	23	17
7	R: supramarginal/angular gyri, superior parietal lobule, inferior parietal lobule	205	4,156	36	–64	47
			3,980	33	–46	44
			3,623	48	–49	44
8	L: supramarginal gyrus, inferior parietal lobule	203	4,139	–33	–49	38
			3,890	–51	–43	53
			3,883	–42	–49	41
Figure selection > RPS game						
1	L: central operculum, supramarginal gyrus, postcentral gyrus, superior temporal gyrus, insula	399	4,773	–45	–16	20
			4,610	–54	–13	17
			4,409	–48	–1	11
2	R: central operculum, supramarginal gyrus, postcentral gyrus, superior temporal gyrus	191	4,661	66	–19	17
			4,343	66	–22	32
			4,130	51	–28	20

R/L, right/left hemisphere; dmPFC, dorsomedial prefrontal cortex; SMA, supplementary motor area.

Conversely, decreased BOLD signal during the RPS game relative to the control condition was observed in the bilateral central operculum, SMG, postcentral gyrus, and superior temporal gyrus, and the left insula.

**FIGURE 3:**
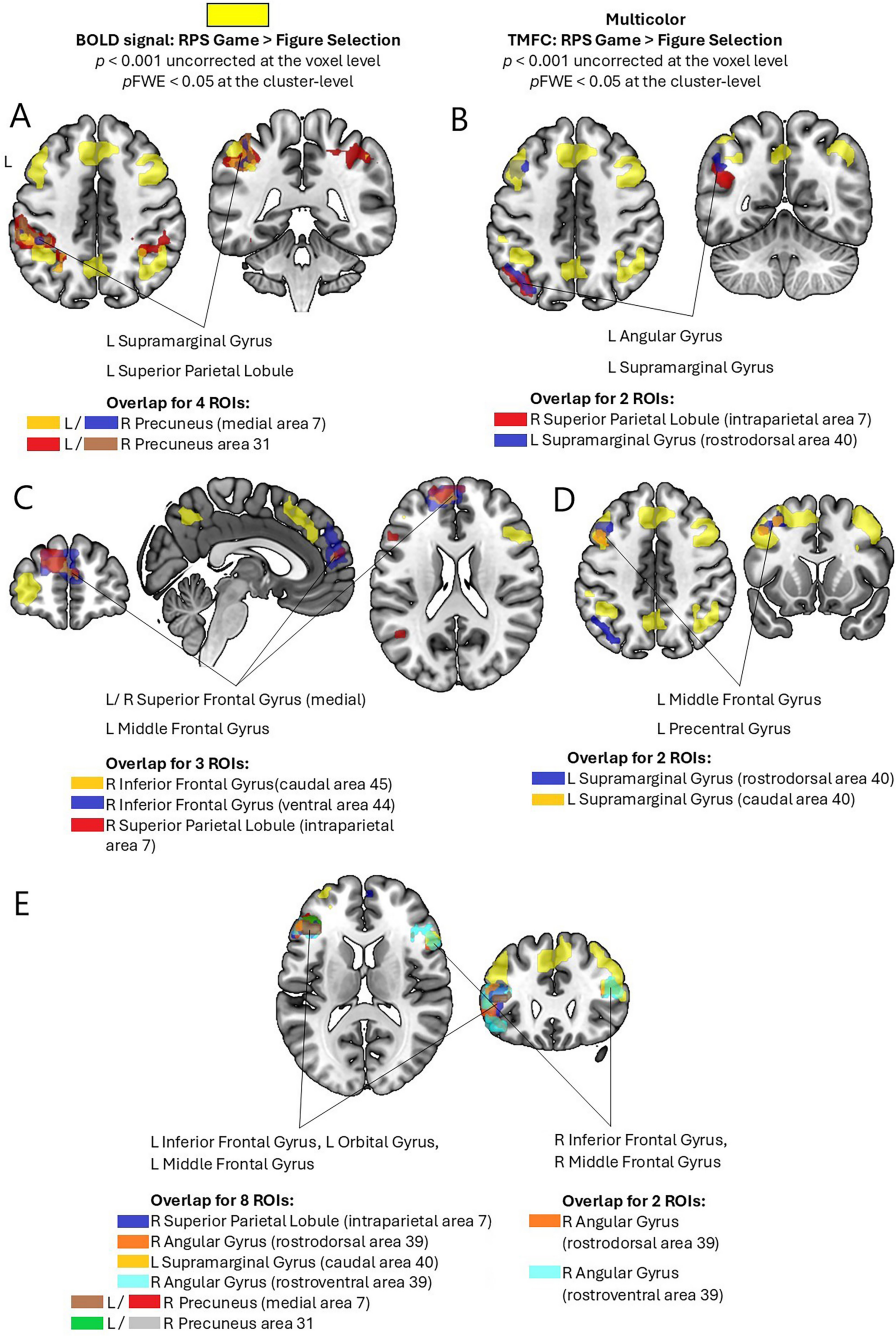
Clusters of overlap in RPS-induced increases in TMFC for the ROIs with RPS-related BOLD signal increases (*p* < 0.001 uncorrected at the voxel level, *pFWE* < 0.05 at the cluster-level). **(A)** Overlap in the left supramarginal gyrus and superior parietal lobule. **(B)** Overlap in the left angular and supramarginal gyri. **(C)** Overlap in the bilateral medial superior frontal gyrus and left middle frontal gyrus. **(D)** Overlap in the left middle frontal and precentral gyri. **(E)** Overlap in the left inferior frontal, orbital, and middle frontal gyri and overlap in the right inferior frontal and middle frontal gyri.

Analysis of RPS-related TMFC revealed several overlapping clusters (see Figure 3, Supplementary Figure 10, and Supplementary Table 3) of task-induced coupling during the RPS condition as compared to the control task, each shared by multiple seed ROIs (see Supplementary Table 4). The first overlapping cluster was observed for ROIs in the bilateral precuneus (medial area 7 and area 31) and encompassed rostral, postcentral, caudal, and intraparietal subdivisions of area 7 in the left SPL, as well as adjacent left SMG regions (rostrodorsal, rostroventral, and caudal area 40). The second overlapping cluster for ROIs in the left SMG (rostrodorsal area 40) and right SPL (intraparietal area 7) was observed in the rostroventral and rostrodorsal subdivisions of area 39 within the left AG and caudal area 40 within the left SMG. The third overlapping cluster was identified for ROIs in the right IFG (caudal area 45 and ventral area 44) and the right SPL (intraparietal area 7). This cluster encompassed medial area 10, medial and lateral subdivisions of area 9 of the bilateral medial superior frontal gyrus, as well as area 46 and dorsolateral area 9/46 in the left middle frontal gyrus. The fourth overlapping cluster was observed for ROIs in the left SMG (rostrodorsal and caudal area 40). This cluster included ventrolateral area 8 and the inferior frontal junction regions of the left middle frontal gyrus, as well as ventrolateral and caudal ventrolateral area 6 in the left precentral gyrus. The fifth overlapping cluster was observed for ROIs located in the right SPL (intraparietal area 7), right AG (rostrodorsal and rostroventral area 39), left SMG (caudal area 40), and bilateral precuneus (medial area 7 and area 31). This cluster encompassed the inferior frontal sulcus, rostral, and caudal area 45 of the left IFG, as well as ventral area 9/46 in the left middle frontal gyrus, and lateral area 12/47 in the left orbital gyrus. The sixth overlapping cluster was identified for ROIs located in the right AG (rostrodorsal and rostroventral area 39). This cluster encompassed dorsal and ventral subdivisions of area 44, caudal area 45, and the inferior frontal sulcus regions of the right IFG, and ventral area 9/46 in the right middle frontal gyrus.

## 4 Discussion

The present study using a modified version of the RPS task successfully reproduced the previously reported increase in BOLD signal within the dorsomedial PFC, right AG and precuneus observed during the RPS game relative to the control figure-selection condition. In addition, we revealed not only right-lateralized but also bilateral supramarginal subregions of the TPJ and bilateral IFG involvement in RPS performance. The observed regions overlap with the ToM neural network according to meta-analytical results (see Figure 2). Behavioral results are compatible with spontaneous opponent modeling during the RPS task utilized in the current study: participants adopted an intentional stance toward the opponent even though the outcomes were preprogrammed and therefore such strategic reasoning did not influence the actual outcome. Therefore, behavioral and fMRI data together are consistent with engagement of the ToM network during RPS performance (Schurz et al., 2021; see Supplementary Data 1.4 for additional behavioral evidence). At the same time, it should be noted that differences in social context, including gaming and stimulus features during the RPS condition, could partially account for the revealed neural differences between the RPS and control tasks, rather than being exclusively associated with the engagement of mentalizing process. Analysis of the direction of BOLD signal changes provided further evidence for the engagement of these brain areas, specifically through greater BOLD increases relative to baseline activity, which is consistent with neural basis of intentional stance adoption rather than non-specific task demands. TMFC results emphasize this point by revealing an increased functional coupling between the ToM network areas, namely, the right IFG with the mPFC and the angular subregion of the right TPJ, as well as of the bilateral TPJ (right angular subregion and left supramarginal subregion) and the precuneus with the left IFG.

Beyond these canonical ToM regions, we identified previously unreported brain structures showing increased activation during RPS compared to the control condition, including the occipital cortex, right frontal operculum, and left FPC. Of particular interest is the FPC (Brodmann area 10), also demonstrating increased functional interactions with the bilateral TPJ and precuneus. It is disproportionately large in humans compared with non-human primates (Semendeferi et al., 2001) and maintains extensive corticocortical connections with multiple prefrontal, posterior parietal, and temporal regions (Mansouri et al., 2017). According to the goal-directed behavior model proposed by Mansouri et al., the FPC participates in evaluating the value of current versus alternative goals and reallocating cognitive resources accordingly. Previous evidence links the FPC region to ToM-related processes: for instance, the extent of Brodmann area 10 lesions correlates with ToM deficits (Roca et al., 2011), and increased activation of the FPC has been observed for ToM-related tasks, being stronger when inferences were made about thoughts, beliefs or intentions compared to emotions (Völlm et al., 2006).

The inclusion of a well-defined control condition also revealed a previously unreported set of regions exhibiting decreased BOLD signal during the RPS game, including the central operculum and left insula. In contrast, prior studies that considered BOLD directionality reported opposite effects—for example, during the task requiring recognition of affective states from eye expressions (Reading the Mind in the Eyes Task/RMET), greater deactivation was found in the same brain regions (mPFC, angular and supramarginal subregions of bilateral TPJ, and precuneus) that in our study demonstrated activation (Zheltyakova et al., 2025). These differences correspond to the well-established dissociation within ToM capacity into two domains: the “affective” (hot) domain, related to the inference of others’ emotions, and the “cognitive” (cold) domain, linked to the attribution of others’ thoughts, intentions, and beliefs (Brothers and Ring, 1992). From this perspective, tasks such as the RMET primarily engage the affective ToM domain, while strategic interaction tasks like the RPS game predominantly recruit the cognitive ToM domain. According to previous meta-analyses, cognitive ToM tasks, when directly compared to affective ToM tasks, elicit stronger activation in the precuneus, bilateral TPJ, right middle temporal gyrus, anterior superior temporal gyrus, and dorsomedial PFC, while the opposite contrast results in stronger activation in the temporal cortex, supplementary motor cortex, and bilateral IFG (Molenberghs et al., 2016; Arioli et al., 2021). Therefore, listed structures with different direction of BOLD signal changes, associated with RMET and RPS tasks, align with the current understanding of the neural basis of the cognitive ToM domain. Further meta-analytic evidence on specific types of ToM tasks demonstrates the increased activation within these regions during false-belief reasoning, personality judgment, and, notably, strategic game tasks, to which the RPS game can be assigned (Schurz et al., 2014, 2021; Molenberghs et al., 2016). This attribution is further supported by a dedicated meta-analysis conducted by Schurz et al. (2021), which examined 13 fMRI studies on strategic games and revealed consistent engagement of the mPFC, precuneus, and right TPJ. Moreover, activations observed during strategic game paradigms were classified within the “cognitive cluster” of ToM neural network, indicating predominant involvement of cognitive mentalizing mechanisms (Schurz et al., 2021). Thus, the neural structures identified in the present study, as well as the nature of their engagement reflected by greater BOLD signal increases, are consistent with current knowledge of both the broader cognitive ToM network and the neural mechanisms underlying strategic interaction tasks such as the RPS game.

The interpretation of observed differences in activation versus deactivation between affective and cognitive ToM tasks may be explained within the dual-pathway model of mentalizing (Kanske et al., 2016; Schurz et al., 2021, 2024). According to this model, the affective pathway depends on attention to external social cues and sensory information, whereas the cognitive pathway relies on internal, abstract mentalizing processes detached from immediate perception and directed inward. A dynamic balance between these inward- and outward-oriented processes was previously connected to integration (increased functional interactions) and segregation (decreased functional interactions) mechanisms (Schurz et al., 2020; Maliske and Kanske, 2022; Kranewitter and Schurz, 2025). Importantly, results obtained both at the level of BOLD signal and TMFC changes for the RPS task suggest the involvement of the integration mechanism during the RPS game. In addition to discussed above areas, referred to the cognitive ToM network subcomponent, playing RPS, compared to the control task, was associated with increased BOLD signal in bilateral IFG, included in the affective ToM network subcomponent. Importantly, increased RPS-related functional interactions were observed between ToM network subcomponents: the bilateral TPJ and precuneus demonstrated increased coupling with the left IFG, while the mPFC and the right TPJ—with the right IFG. Building on this, our findings suggest that the task type influences the nature of engagement of ToM-related brain regions, expressed not only through changes in functional interactions but also through stronger decreases or increases in BOLD signal reflecting difference in ToM demands.

In conclusion, the present study extends current knowledge of the neural organization of the ToM network by demonstrating that engagement of key ToM regions—the mPFC, TPJ, and precuneus—during social interaction depends on task demands and manifests through directionally distinct BOLD responses. These findings indicate that both cognitive and affective domains of ToM, recruited during strategic interactions such as the RPS game, are characterized by increased BOLD activity in classical ToM-associated brain areas. TMFC results further support the integration between cognitive and affective ToM network subcomponents associated with strategic interactions. In contrast, some of these regions were previously connected to BOLD signal suppression below baseline during tasks engaging the affective ToM domain (i.e., RMET), reflecting a shift toward externally oriented processing. Future research combining connectivity analyses and cross-task comparisons is warranted to further elucidate how different brain regions of ToM network flexibly transition between affective and cognitive aspects of social cognition.

## Data Availability

The raw data supporting the conclusions of this article will be made available by the authors, without undue reservation.

## Footnotes

1
http://www.fil.ion.ucl.ac.uk/spm

2
https://github.com/IHB-IBR-department/TMFC_toolbox
