# Supplementary material for: Task-dependent increases and decreases of BOLD signal in theory of mind brain regions during strategic social interaction
Source: Front Neural Circuits. 2026 Apr 8;20:1741762. doi: 10.3389/fncir.2026.1741762 (PMC13099858; doi:10.3389/fncir.2026.1741762)
Supplement: Supplementary file 1 [file Data_Sheet_1.docx]

Supplementary Material

# Supplementary Data

## Experimental Task

For the RPS game participants received instructions to select the option that they thought would allow them to win against the opponent. In each RPS game trial, participants freely selected one of three gestures (rock, paper, or scissors) using corresponding button presses (see Figure 1B). Rock always appeared in the left position, paper – in the center, and scissors – in the right. After each selection, feedback including participant’s choice, opponent’s choice, and the trial outcome was displayed (“You won,” “You lost,” “Draw,” or “No response”). Therefore, as in real-life gameplay, participants could infer the opponent’s intentions and strategy from prior choices, predict future actions, and adjust their own decisions accordingly.

The control condition required participants to select the correct figure from the three geometric shapes (triangle, square, or circle) presented on the screen (see Figure 1C). The experiment consisted of three sessions, each corresponding to one of the figures; the order of sessions was counterbalanced across participants. Before the beginning of each session participants were verbally instructed as to which one of the figures is the correct one. All three figures appeared in the center of the screen in a row, and the task was to press the button corresponding to the left, right, or middle position of the target figure on the screen. In each control trial the position of the correct figure on the screen appeared randomly. As in the RPS condition, feedback was provided after each trial (“Correct,” “Incorrect,” or “No response”).

## Response Fixation

To select the gesture in the RPS game and to pick the correct figure in the figure selection task participants used two MR-compatible controllers held in both hands with two buttons on each. There were three variants of association between the button and the position on screen, counterbalanced across participants: 1) left thumb – left position, left index finger – center position, right index finger – right position; 2) left thumb – left position, right index finger – center position, right thumb – right position; 3) left index finger – left position, right index finger – center position, right thumb – right position.

## Task-Modulated Functional Connectivity Analysis

### Selection of regions of interest

In accordance with widely used approach, regions of interests for the functional connectivity analysis are selected based on the results of task-activation analysis using local maxima of the group statistical assessment of the BOLD signal difference between compared conditions. In some cases, it is allowed to move these individual local maximа within the predefined area surrounding group local maxima. Although the latter allows to increase the sensitivity of TMFC analysis, in both cases the anatomical precision of ROI location is low, especially when large ROIs are used. This could potentially underestimate anatomical information, which is particularly important for the key ToM network regions like temporoparietal junction. To address this issue, ROIs were defined by finding the overlap between clusters of BOLD signal changes revealed on the stage of activation analysis and the Brainnettom atlas (Fan et al., 2016). Resulted ROIs, for which the significant functional connectivity changes were obtained are illustrated in Supplementary Figure 1.

### Statistical Analysis

Functional connectivity analysis was performed using task modulated function connectivity (TMFC) and TMFC toolbox (https://github.com/IHB-IBR-department/TMFC_toolbox) in the ROI-to-seed manner. Beta-series correlation least-squares separate approach (BSC-LSS) was applied since this is more sensitive and specific method for the event-related fMRI design (Mumford et al., 2012; Masharipov et al, 2024). To improve the sensitivity of TMFC analysis the co-activations were controlled by finite impulse response (FIR) task regression procedure (for details see Masharipov et al, 2024). During the individual BSC-LSS analysis, a separate GLM was estimated for each trial, including one regressor for the trial of interest and one regressor for all other trials. Modeled conditions of interest were the same as for activation analysis (“RPS Game” and “Figure Selection” trials), whereas feedback trials and trials without response were treated as conditions of no interest. Durations of modeled conditions were the same as for the BOLD signal changes analysis. Twenty-four motion parameters were included as nuisance regressors. Such analysis was performed for each selected ROI.

At the group level of analysis, one-sample *t*-tests were applied for the “RPS Game > Figure Selection” contrast of TMFC betas taken from individual analyses. Although the effect of scanner was not observed for the BOLD signal changes analysis, this effect was revealed for the TMFC estimates. Therefore, to account for scanner difference the covariates modeling the effect of scanner were introduced to the one-sample *t*-test GLM model.

Statistical inference for TMFC analysis was performed using classical frequentist statistics with a significance threshold of *p* < 0.001 uncorrected at the voxel level, followed by cluster-level FWE correction at *p* < 0.05. A gray matter mask derived from individual T1 segmentation maps was applied to restrict analyses to brain tissue.

### Data Visualization

The XjView Toolbox (https://www.alivelearn.net/xjview/) was used to determine the anatomical location of significant clusters, and MRIcroGL (<https://www.nitrc.org/projects/mricrogl/>) was used for visualization and figure generation.

## Linking RPS-related neural activity to mentalizing processes

In the current RPS experimental design opponent’s responses were programmed to provide fixed ratio of wins, losses, and draws for all participants. This creates the concern that mentalizing was neither necessary nor instrumentally beneficial for performance. Under this interpretation, neural differences between RPS and the control task could reflect gaming context, or stimulus-response features rather than engagement of mentalizing processes per se. The interpretation is complicated by the fact that an activity such as playing a strategic game like RPS includes multiple interwoven processes that are not always separable from one another. However, we can still draw conclusions and justify the linkage of the obtained results specifically to mentalizing by (1) comparing them with the literature and (2) providing behavioral data evidencing towards hypothesized prediction of the opponent's choices during RPS game.

### Literature Comparison

The RPS game is believed to induce a specific component of **online** mentalizing – the adoption of an intentional stance (Gallagher et al., 2002; Chaminade et al., 2012). It has been argued that interactive game settings **require** participants to make assumptions about an opponent’s beliefs, desires, and intentions **in order to** guide their future actions in the game and to win. The involvement of **intentional stance adoption** was further confirmed by post-experimental questionnaires, according to which participants **reported conceiving** of their opponent as a rational agent after **being verbally instructed** that they would play against another human (Gallagher et al., 2002). **Consequently,** the selected experimental design is **grounded in** inferring others’ intentions, and the procedure, where participants were instructed that they were playing against a real opponent, induced mentalizing. In support, BOLD signal increases observed in the RPS condition compared to the control condition comprised multiple regions that, as demonstrated by their overlap with meta-analytical results, comprise the ToM neural network associated with mentalizing (Schurz et al., 2021) (Figure 2).

On the contrary, previous studies that used the RPS game to investigate processes other than mentalizing reported BOLD signal changes in distinct brain areas. In particular, a study on cognitive control linked increased BOLD signal in the left dorsolateral prefrontal cortex to conditions in which participants were required to lose (compared to the standard instruction to win), interpreting this effect as reflecting inhibition of stereotyped responses during the game. Transcranial magnetic stimulation (TMS) delivered to this area also led to increased response errors (i.e., erroneous wins when players were required to lose) (Kadota et al., 2010). Another study using RPS and applying multivoxel pattern analysis (MVPA) showed that signals related to reinforcement and punishment during the feedback phase are widely distributed across nearly all subdivisions of the human brain (Vickery et al., 2011).

Additional line of support is that brain areas including the mPFC, precuneus, and parts of the TPJ also partially overlap anatomically with the default mode network (DMN) (Schilbach et al., 2008, 2012; Yeo et al., 2011), typically engaged in internally directed cognition and reducing activity during externally oriented or goal-directed tasks (Raichle et al., 2001, 2015). If the obtained results were associated with the general cognitive control (and not with processes specifically induced by the RPS game, such as mentalizing), activity in the named areas should have been reduced during the more complex task. However, although response times were numerically different, this difference did not reach statistical significance in comparison between the RPS (947 ± 290 ms) and control conditions (880 ± 187 ms). Therefore, the observed BOLD signal increase in the mPFC, precuneus, and the TPJ, is consistent with the involvement of specific task-related processes rather than general effect of task complexity.

### Behavioral Evidence for Opponent Modeling During RPS

To directly address the issue, we tested whether RPS performance reflected mentalizing rather than simple reinforcement learning (RL), namely, we examined whether participants’ choice sequences exhibited signatures of opponent modeling that cannot be explained by random play or first-order RL.

In mixed-strategy games such as RPS, randomization (*P* = 1/3 per gesture) is optimal only against an unexploitable opponent. Against a fallible agent, however, success requires the opposition to randomization – detecting and anticipating regularities in the opponents’ behavior in order to outwit them (Brockbank and Vul, 2021, 2024).

Pure RL strategies are based on win-stay/lose-shift strategy (WSLS) and are exploitable because they generate predictable outcome-dependent responses (Sundvall and Dyson, 2022). This strategy is employed by primates to play against simple algorithms (Barraclough et al., 2004; Seo et al., 2007) or by humans to play against a computer algorithm (Vickery et al., 2011; Dyson et al., 2016). In contrast, deviation from RL, particularly outcome-independent switching and higher-order sequential structure, has been interpreted as evidence for elementary mentalizing in competitive games (Hampton et al., 2008; Hill et al., 2017).

We therefore identified three converging behavioral signatures that collectively demonstrate possible involvement of mentalizing during the decision phase, before outcome feedback: (i) active repetition avoidance, (ii) reversal of the canonical win-stay effect, and (iii) second-order sequential structure (lag-2 effects). Across all metrics, participants systematically deviated from RL predictions.

#### Active Repetition Avoidance

The simplest exploitable response pattern is self-repetition (Dyson, 2019). Whereas RL predicts elevated repetition via win-stay, mentalizing predicts active suppression to avoid predictability. The observed repetition rate (*M* = 0.274, *SD* = 0.087) was significantly below the random baseline of 1/3, *t*(45) = −4.63, *p* < .001, *d* = −0.68; 89% of participants fell below chance. This anti-repetition bias indicates deliberate unpredictability rather than reinforcement-driven persistence.


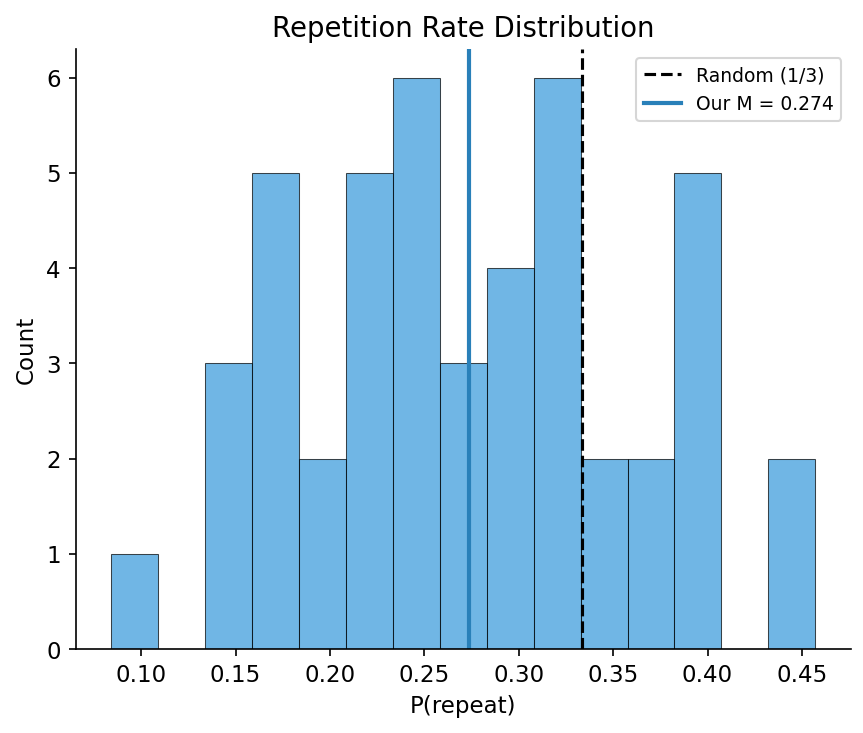


**Supplementary Figure 1. Repetition rate distribution.** The histogram shows the distribution of the repetition avoidance scores across participants, showing the count of participants within each bin. The blue line marks the group mean, and the dashed black line indicates the random baseline (0.33).

#### Anti-Win-Stay (Reversed RL)

Standard RL predicts robust win-stay behavior in standard RPS (≈0.40 in typical RPS; Dyson et al., 2016). Instead, all stay rates were below 1/3, including after wins (*P*(stay|Win) = 0.258, *t*(45) = −4.67, *p* < .001) (Supplementary Figure 2, Supplementary Table 1**)**. Thus, participants showed the opposite of RL: they systematically avoided repeating a winning gesture. Such reversal is consistent with hypothesized opponent modeling (“the opponent will adapt to my win”) and incompatible with both RL (>1/3) and random responding (=1/3).


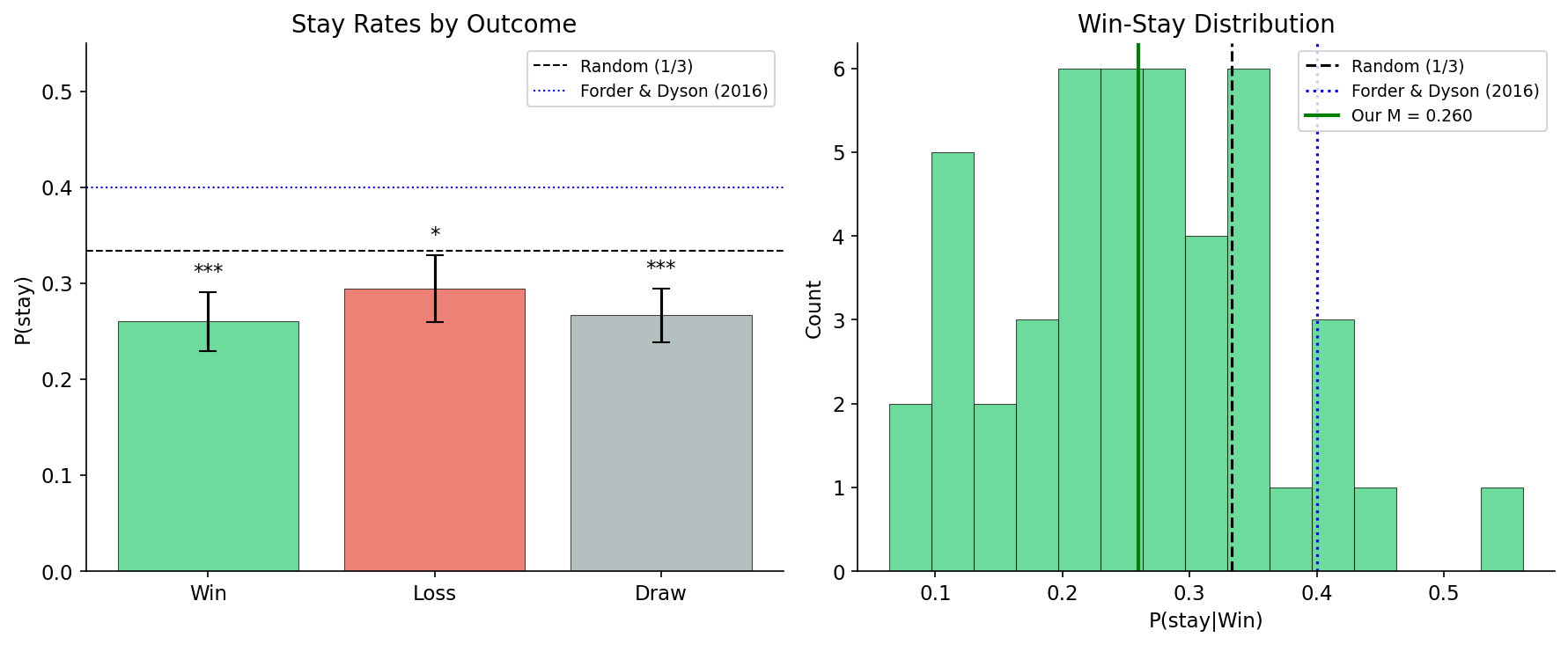


**Supplementary Figure 2. Win-stay behavior by outcome type.** The bar plot on the left displays group means and 95% CI for the probability of repeating the previous choice (*P*(stay)) as a function of the preceding outcome (Win, Loss, or Draw). The dashed horizontal black line indicates the random baseline (0.33), and the dotted blue line represents the reference *P*(stay|Win) value reported by Forder et al. (2016). The histogram on the right illustrates the distribution of win–stay behavior across participants, showing the count of participants within each bin. The green line marks the group mean, the dashed black line indicates the random baseline (0.33), and the dotted blue line represents the reference value from Forder et al. (2016).

**Supplementary Table 1. Win-stay behavior by outcome type.** The probability of repeating the previous choice (*P*(stay)) depending on the preceding outcome (Win, Loss, or Draw)

| **Outcome** | ***P*(stay)** | ***t*(45)** | ***p*** | **Cohen's *d*** |
| --- | --- | --- | --- | --- |
| **After Win** | **0.258** | −4.67 | < .001 | **−0.69** |
| After Draw | 0.264 | −4.68 | < .001 | −0.69 |
| After Loss | 0.293 | −2.21 | .032 | −0.33 |

#### Outcome Independence

A defining feature of RL is strong outcome modulation, which typically yields *P*(stay|Win) − *P*(stay|Loss) ≈ +0.20 (Dyson et al., 2016). In the current study, this difference was −0.034 (*p* = .085), which is not significantly different from zero and numerically reversed (Supplementary Figure 3). Participants switched approximately 73% of the time regardless of outcome, demonstrating outcome-independent strategy selection. This contradicts the prediction based on RL but is consistent with mentalizing accounts in which anticipated opponent adaptation dominates reward history.


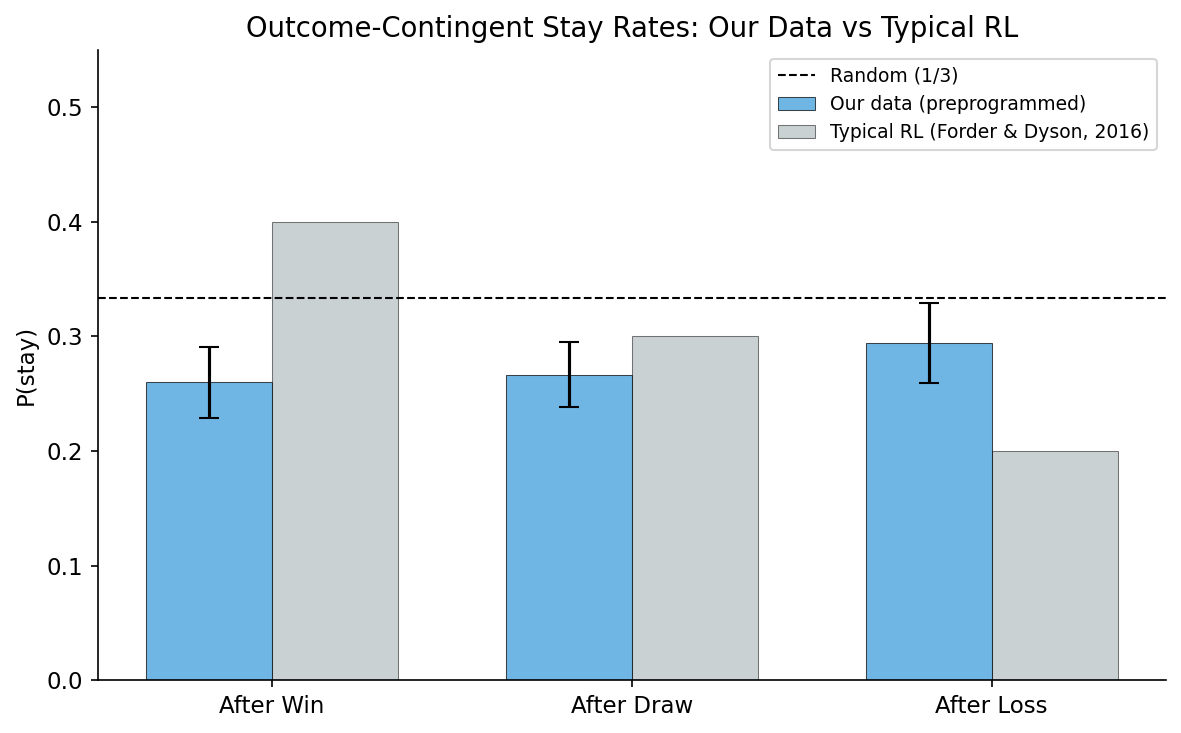


**Supplementary Figure 3. Stay rates by outcome type.** The blue bars display group means and 95% CI for the probability of repeating the previous choice (*P*(stay)) as a function of the preceding outcome (Win, Loss, or Draw). The dashed horizontal black line indicates the random baseline (0.33), and the grey bars represent the reference (*P*(stay)) value as a function of the preceding outcome (Win, Loss, or Draw) reported by Forder et al. (2016) for a typical reinforcement learning pattern.

#### Forward Cycling (Second-Order Structure)

First-order RL cannot generate structured dependencies beyond the immediately preceding trial. When two consecutive switches occurred (A→B→?), RL predicts equal probability of returning to A or cycling forward to C (0.50). The observed lag-2 return rate (*M* = 0.444, *SD* = 0.090) was significantly below 0.50, *t*(45) = −4.23, *p* < .001, *d* = −0.62, indicating systematic forward cycling (Supplementary Figure 4). Such second-order structure is consistent with iterative reasoning models of strategic interaction (Crawford et al., 2013; Wang et al., 2014; Dyson, 2019) and influence-based learning frameworks (Hampton et al., 2008), which require modeling how one’s own actions shape the opponent’s expectations.


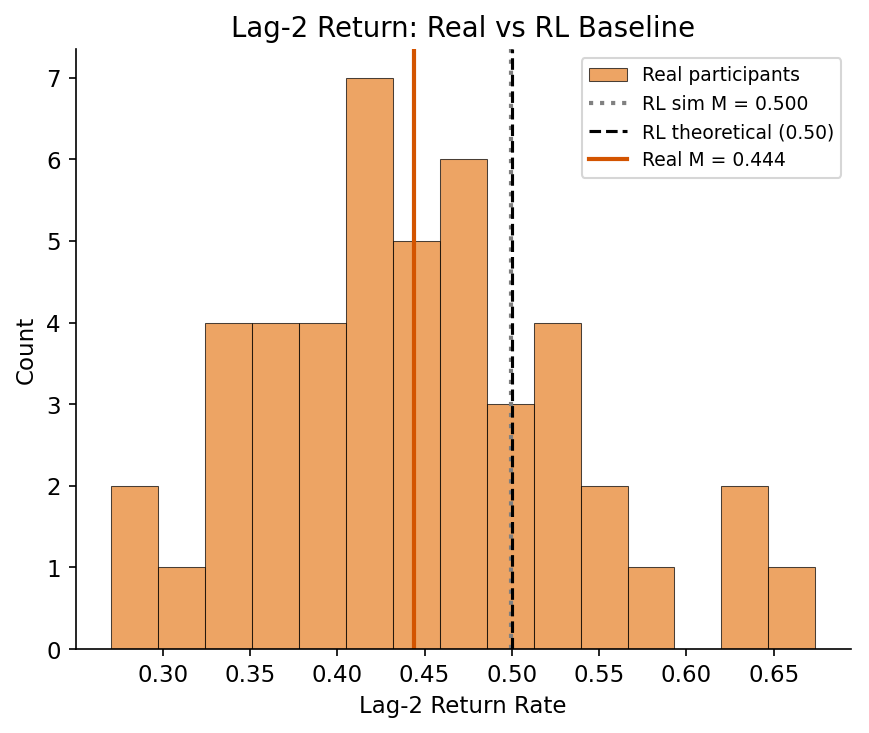


**Supplementary Figure 4. Lag-2 return rate.** The histogram on the left shows the distribution of the lag-2 return rate across participants, showing the number of participants within each bin. The red line marks the group mean, the dashed black line indicates the random lag-2 return rate baseline (0.5), and the dotted grey line represents the lag-2 return rate simulated from 1,000 RL agents (0.5).

#### The Critical RL Simulation Test

To rule out RL more definitively, we simulated 1,000 RL agents per participant matched exactly to each individual’s empirical outcome-contingent stay rates and exposed them to the identical outcome sequence. By construction, simulated agents reproduced all first-order statistics (entropy, repetition, runs, longest run, cycling direction, *p* > .15 for all). Crucially, however, they failed to reproduce the reduced lag-2 return rate observed in real participants (*z* = −1.05, *t*(45) = −4.68, *p* < .001, *d* = −0.69) (Supplementary Figure 5). Thus, higher-order sequential structure cannot be reduced to first-order RL.


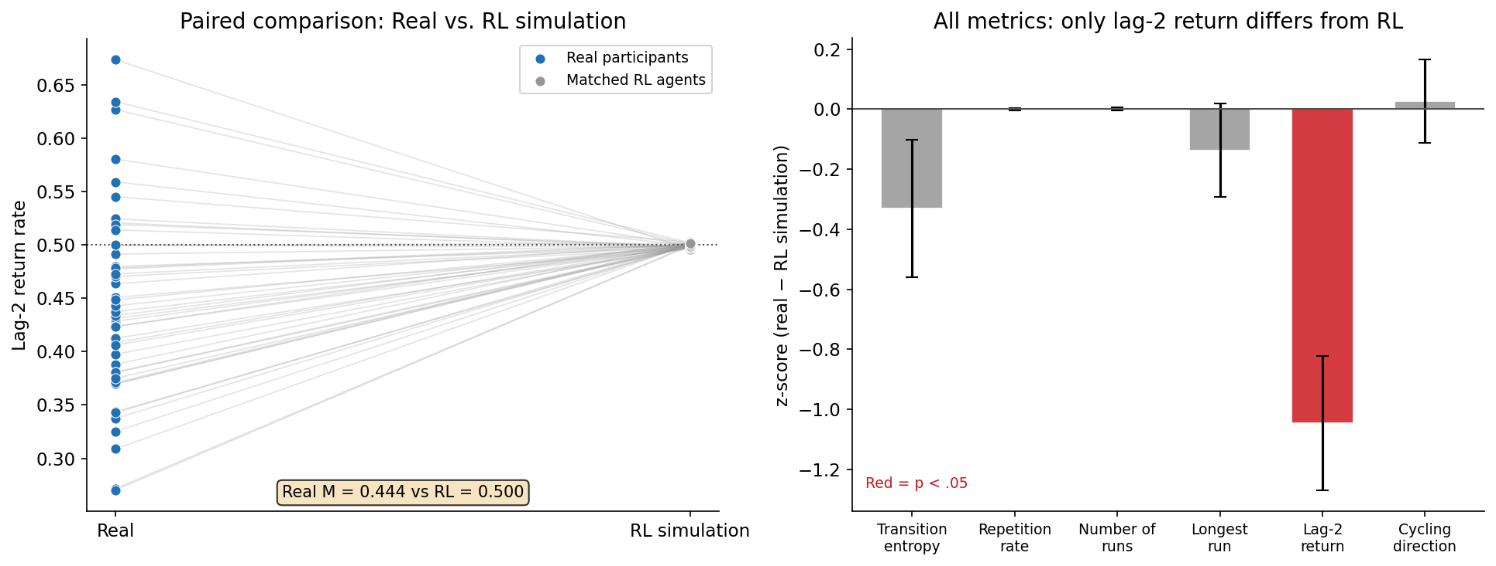


**Supplementary Figure 5. Comparison between real and simulated data.** The panel on the left presents a paired comparison of observed individual lag-2 return rates (blue point, one per participant) and the reference lag-2 return rate simulated from 1,000 RL agents (grey point).The bar plot on the right displays group means and 95% CI for first-order statistics (transition entropy, repetition rate, number of runs, longest run, cycling direction) and second-order statistics (lag-2 return rate). Red bar represents significant deviation of the observed lag-2 return rate from the value simulated from 1,000 RL agents (*p* < .001, Cohen’s *d* = −0.69).

#### Transition Matrix Control

Forward cycling did not reflect a global directional bias (e.g., Rock→Paper→Scissors). Off-diagonal transition probabilities were symmetric (*P*(upward) = 0.499, *p* = .90), indicating that the effect was local (avoidance of returning to t−2) rather than a fixed motor or directional preference (Supplementary Figure 6).


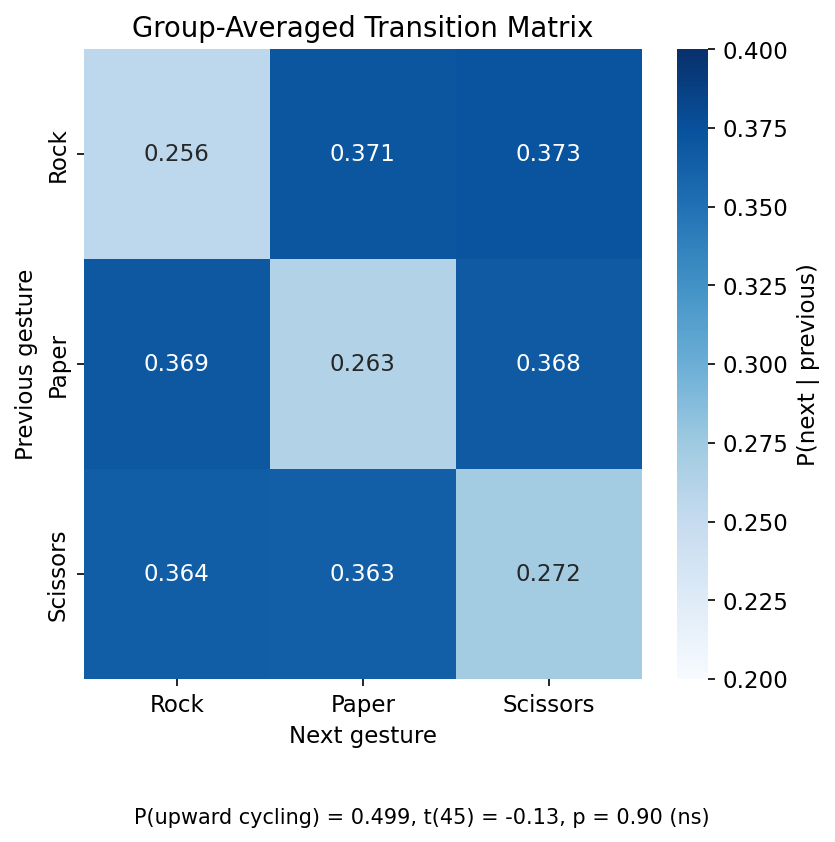


**Supplementary Figure 6. Transition probability matrix between gestures.**
The heatmap displays the probability of transitioning from one gesture (row) to another (column) in the next trial. Values represent the conditional probability $P(\text{choice}_{t+1}\mid\text{choice}_{t})$ for each pair of gestures (Rock, Paper, Scissors). The diagonal entries (staying with the same gesture) are notably below chance (0.33) for all gestures. Off-diagonal probabilities are generally elevated and symmetric.

#### Convergent Validity

Indices of anti-repetition, anti-win-stay, and forward cycling were positively and significantly correlated, supporting a common underlying mechanism rather than independent heuristics (Supplementary Table 2 and Supplementary Figure 7).


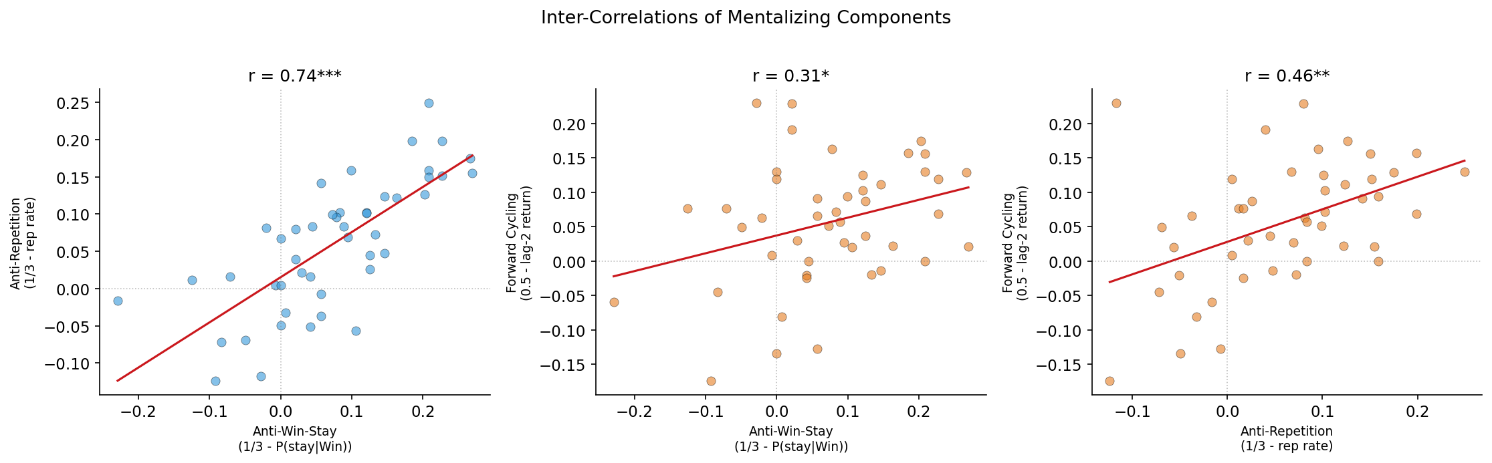


**Supplementary Figure 7. Inter-correlations of mentalising components.** The panel on the left shows the relationship between win-stay behavior and repetition avoidance. The middle panel shows the relationship between win–stay behavior and forward cycling. The panel on the right shows the relationship between win-stay behavior and repetition avoidance. Each point in scatterplots represents an individual participant.

**Supplementary Table 2.** Pairwise correlations between key behavioral signatures indicative of opponent modeling

| Pair | *r* | *p* |
| --- | --- | --- |
| Anti-win-stay ↔ Anti-repetition | +0.75 | < .0001 |
| Anti-win-stay ↔ Forward cycling | +0.31 | .037 |
| Anti-repetition ↔ Forward cycling | +0.46 | .001 |

### Conclusion

In conclusion, the consideration of literature provides partial support for interpreting the obtained results as being related to mentalizing. To further substantiate this interpretation, the behavioral data demonstrates that participants adopted strategies characteristic of opponent modeling during RPS game (Supplementary Figure 8). The presence of active repetition avoidance, reversal of canonical win-stay effects, outcome independence, and second-order sequential structure, the latter of which was not reproduced in the simulation study with the usage of matched RL agents or explained by fixed motor or directional preference. Thus, abovementioned behavioral indices collectively evidenced towards possible involvement of processes beyond reward tracking or randomization and linked observed responding pattern to mentalizing-related process of prediction of the opponent's choices.


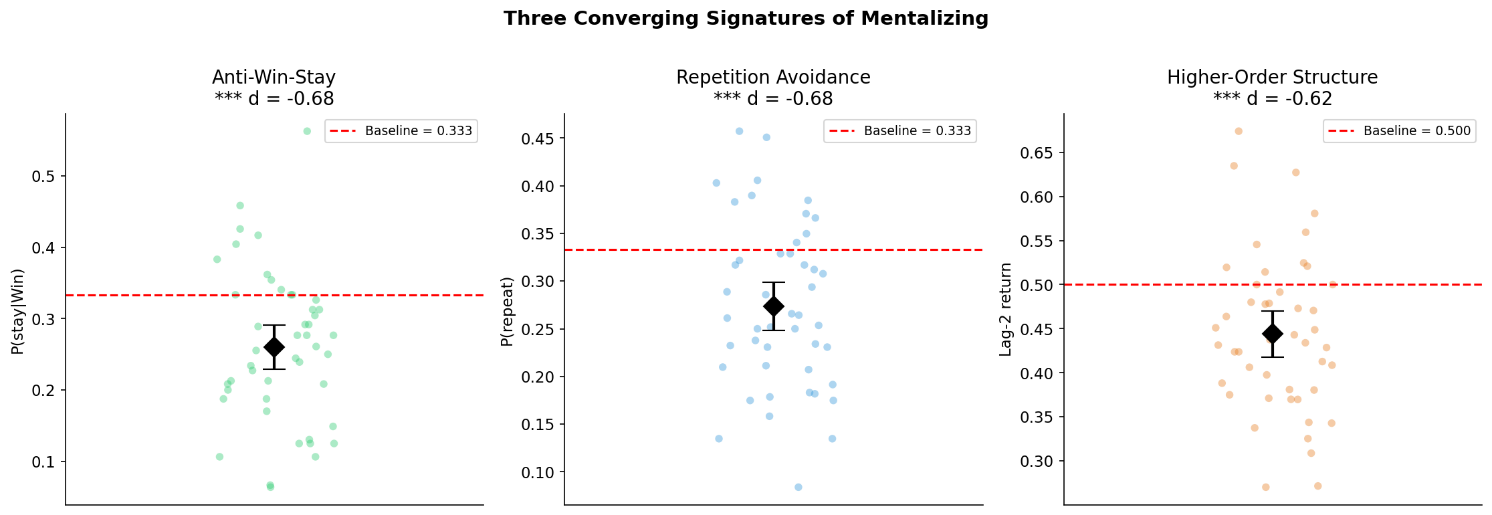
**Supplementary Figure 8**. Three converging behavioral signatures of mentalizing. Individual data points with group means (diamond) and 95% CI for anti-win-stay (*P*(stay|Win) vs. 1/3), repetition avoidance (repetition rate vs. 1/3), and forward cycling (lag-2 return vs. 0.50). All signatures significantly deviate from their respective baselines (dashed red lines), with medium-to-large effect sizes (|*d*| = 0.62–0.69). *** *p* < .001.

# Supplementary Figures and Tables

**Supplementary Table 3.** Clusters of overlap in RPS-induced increases in TMFC for the ROIs with RPS-related BOLD signal increases (*p* < 0.001 uncorrected at the voxel level, *pFWE* < 0.05 at the cluster-level).

| Brain areas | ROI (BNT Nаme) | Brain area (BNT) | x | y | z | *pFWE* clust | cluster size |
| --- | --- | --- | --- | --- | --- | --- | --- |
| L Supramarginal Gyrus  L Superior Parietal Lobule | L Precuneus  medial area 7 | rostrodorsal area 40;  rostroventral area 40;  caudal area 40;  rostral area 7;  postcentral area 7 | -48 | -37 | 38 | 0.000 | 316 |
|  |  |  | -27 | -58 | 53 |  |  |
|  | R Precuneus  medial area 7 | rostrodorsal area 40 | -45 | -37 | 38 | 0.051 | 68 |
|  |  |  | -42 | -46 | 62 |  |  |
|  |  |  | -60 | -28 | 35 |  |  |
|  | L Precuneus area 31 | rostrodorsal area 40;  rostroventral area 40;  caudal area 40;  rostral area 7;  caudal area 7;  intraparietal area 7 | -57 | -34 | 44 | 0.000 | 795 |
|  |  |  | -57 | -16 | 29 |  |  |
|  |  |  | -51 | -37 | 38 |  |  |
|  | R Precuneus area 31 | rostrodorsal area 40;  rostroventral area 40;  caudal area 40;  rostral area 7;  postcentral area 7 | -54 | -28 | 56 | 0.000 | 300 |
|  |  |  | -57 | -16 | 29 |  |  |
|  |  |  | -42 | -37 | 47 |  |  |
| L Angular Gyrus  L Supramarginal Gyrus | R Superior Parietal Lobule  intraparietal area 7 | rostroventral area 39;  rostrodorsal area 39;  caudal area 40 | -45 | -55 | 26 | 0.000 | 207 |
|  |  |  | -39 | -76 | 38 |  |  |
|  |  |  | -51 | -64 | 32 |  |  |
|  | L Supramarginal Gyrus  rostrodorsal area 40 | rostroventral area 39;  rostrodorsal area 39;  caudal area 40 | -51 | -58 | 38 | 0.021 | 87 |
|  |  |  | -36 | -73 | 44 |  |  |
| L/ R Superior Frontal Gyrus (medial)  L Middle Frontal Gyrus | R Inferior Frontal Gyrus  caudal area 45 | medial area 10 | -12 | 53 | 20 | 0.039 | 74 |
|  |  |  | -6 | 59 | 23 |  |  |
|  | R Inferior Frontal Gyrus  ventral area 44 | medial area 10;  medial area 9;  lateral area 9;  area 46;  dorsolateral area 9/46 | -3 | 59 | 20 | 0.000 | 353 |
|  |  |  | -12 | 53 | 20 |  |  |
|  |  |  | 15 | 38 | 38 |  |  |
|  | R Superior Parietal Lobule  intraparietal area 7 | lateral area 9;  medial area 10;  area 46; | -12 | 56 | 20 | 0.005 | 117 |
|  |  |  | -18 | 56 | 29 |  |  |
|  |  |  | 6 | 59 | 17 |  |  |
| L Middle Frontal Gyrus  L Precentral Gyrus | L Supramarginal Gyrus  rostrodorsal area 40 | ventrolateral area 8;  inferior frontal junction | -33 | 17 | 50 | 0.014 | 96 |
|  |  |  | -42 | 17 | 47 |  |  |
|  |  |  | -36 | 23 | 35 |  |  |
|  | L Supramarginal Gyrus  caudal area 40 | ventrolateral area 8;  inferior frontal junction;  ventrolateral area 6;  caudal ventrolateral area 6 | -33 | 17 | 50 | 0.042 | 74 |
|  |  |  | -48 | 11 | 44 |  |  |
|  |  |  | -42 | 5 | 50 |  |  |
| L Inferior Frontal Gyrus  L Orbital Gyrus  L Middle Frontal Gyrus | R Superior Parietal Lobule  intraparietal area 7 | caudal area 45;  inferior frontal sulcus | -51 | 29 | 8 | 0.004 | 124 |
|  |  |  | -57 | 23 | 8 |  |  |
|  |  |  | -48 | 26 | -4 |  |  |
|  | R Angular Gyrus  rostrodorsal area 39 | caudal area 45;  inferior frontal sulcus;  rostral area 45;  lateral area 12/47 | -54 | 29 | 8 | 0.004 | 128 |
|  |  |  | -48 | 29 | -4 |  |  |
|  |  |  | -54 | 23 | 20 |  |  |
|  | L Supramarginal Gyrus  caudal area 40 | caudal area 45;  inferior frontal sulcus;  ventral area 9/46 | -42 | 50 | -1 | 0.030 | 82 |
|  |  |  | -51 | 32 | 11 |  |  |
|  | R Angular Gyrus  rostroventral area 39 | caudal area 45;  inferior frontal sulcus;  lateral area 12/47 | -54 | 32 | 11 | 0.000 | 211 |
|  |  |  | -42 | 26 | -10 |  |  |
|  |  |  | -54 | 23 | 20 |  |  |
|  | L Precuneus  medial area 7 | inferior frontal sulcus  caudal area 45  ventral area 9/46 | -39 | 35 | 11 | 0.002 | 156 |
|  |  |  | -42 | 32 | 23 |  |  |
|  | R Precuneus  medial area 7 | inferior frontal sulcus;  caudal area 45;  ventral area 9/46 | -42 | 29 | 11 | 0.002 | 152 |
|  |  |  | -39 | 35 | 26 |  |  |
|  |  |  | -39 | 17 | 23 |  |  |
|  | L Precuneus area 31 | inferior frontal sulcus;  caudal area 45;  ventral area 9/46 | -48 | 29 | 11 | 0.002 | 152 |
|  |  |  | -48 | 38 | 8 |  |  |
|  |  |  | -39 | 35 | 11 |  |  |
|  | R Precuneus area 31 | inferior frontal sulcus;  caudal area 45 | -48 | 29 | 11 | 0.016 | 91 |
|  |  |  | -39 | 29 | 11 |  |  |
|  |  |  | -33 | 29 | 23 |  |  |
| R Inferior Frontal Gyrus  R Middle Frontal Gyrus | R Angular Gyrus  rostrodorsal area 39 | dorsal area 44;  inferior frontal sulcus;  ventral area 44;  ventral area 9/46 | 48 | 23 | 23 | 0.034 | 76 |
|  |  |  | 57 | 17 | 14 |  |  |
|  |  |  | 39 | 23 | 14 |  |  |
|  | R Angular Gyrus  rostroventral area 39 | dorsal area 44;  caudal area 45;  inferior frontal sulcus;  ventral area 44;  ventral area 9/46 | 57 | 17 | 14 | 0.001 | 171 |
|  |  |  | 48 | 23 | 29 |  |  |
|  |  |  | 51 | 29 | 17 |  |  |

R/L, right/left hemisphere

**Supplementary Table 4.** RPS-induced increases in TMFC as compared with control condition (*p* < 0.001 uncorrected at the voxel level, *pFWE* < 0.05 at the cluster-level).

| **Brain areas** | **Brain area (BNT)** | **x** | **y** | **z** | ***pFWE* clust** | **cluster size** |
| --- | --- | --- | --- | --- | --- | --- |
| **ROI in the right middle fontal gyrus (ventral area 9/46)** | | | | | | |
| R Angular Gyrus | rostrodorsal area 39;  rostroventral area 39 | 42 | -61 | 47 | 0.005 | 135 |
| **ROI in the right inferior frontal gyrus (caudal area 45)** | | | | | | |
| L Superior Frontal Gyrus (medial) | medial area 10 | -12 | 53 | 20 | 0.039 | 74 |
|  |  | -6 | 59 | 23 |  |  |
|  |  | 3 | 56 | 14 |  |  |
| **ROI in the right inferior frontal gyrus (ventral area 44)** | | | | | | |
| L/R Superior Frontal Gyrus (medial)  L Middle Frontal Gyrus | medial area 10;  medial area 9;  lateral area 9;  area 46;  dorsolateral area 9/46 | -3 | 59 | 20 | 0.000 | 353 |
|  |  | -12 | 53 | 20 |  |  |
|  |  | 15 | 38 | 38 |  |  |
| **ROI in the right superior parietal lobule (intraparietal area 7)** | | | | | | |
| L Angular Gyrus  L Supramarginal Gyrus | rostroventral area 39;  rostrodorsal area 39;  caudal area 40 | -45 | -55 | 26 | 0.000 | 207 |
|  |  | -39 | -76 | 38 |  |  |
|  |  | -51 | -64 | 32 |  |  |
| L Inferior Frontal Gyrus | caudal area 45;  inferior frontal sulcus | -51 | 29 | 8 | 0.004 | 124 |
|  |  | -57 | 23 | 8 |  |  |
|  |  | -48 | 26 | -4 |  |  |
| L/R Superior Frontal Gyrus (medial)  L Middle Frontal Gyrus | lateral area 9;  medial area 10;  area 46 | -12 | 56 | 20 | 0.005 | 117 |
|  |  | -18 | 56 | 29 |  |  |
|  |  | 6 | 59 | 17 |  |  |
| **ROI in the right angular gyrus (rostrodorsal area 39)** | | | | | | |
| L Inferior Frontal Gyrus  L Orbital Gyrus | caudal area 45;  inferior frontal sulcus;  rostral area 45;  lateral area 12/47 | -54 | 29 | 8 | 0.004 | 128 |
|  |  | -48 | 29 | -4 |  |  |
|  |  | -54 | 23 | 20 |  |  |
| R Inferior Frontal Gyrus  R Middle Frontal Gyrus | dorsal area 44;  inferior frontal sulcus;  ventral area 44;  ventral area 9/46 | 48 | 23 | 23 | 0.034 | 76 |
|  |  | 57 | 17 | 14 |  |  |
|  |  | 39 | 23 | 14 |  |  |
| **ROI in the left supramarginal gyrus (rostrodorsal area 40)** | | | | | | |
| L Middle Frontal Gyrus | ventrolateral area 8;  inferior frontal junction | -33 | 17 | 50 | 0.014 | 96 |
|  |  | -42 | 17 | 47 |  |  |
|  |  | -36 | 23 | 35 |  |  |
| L Angular gyrus  L Supramarginal Gyrus | rostroventral area 39;  rostrodorsal area 39;  caudal area 40 | -51 | -58 | 38 | 0.021 | 87 |
|  |  | -36 | -73 | 44 |  |  |
| **ROI in the left supramarginal gyrus (caudal area 40)** | | | | | | |
| L Middle Frontal Gyrus;  L Precentral Gyrus | ventrolateral area 8;  inferior frontal junction;  ventrolateral area 6;  caudal ventrolateral area 6 | -33 | 17 | 50 | 0.042 | 74 |
|  |  | -48 | 11 | 44 |  |  |
|  |  | -42 | 5 | 50 |  |  |
| L Inferior Frontal Gyrus  L Middle Frontal Gyrus | caudal area 45;  inferior frontal sulcus;  ventral area 9/46 | -42 | 50 | -1 | 0.030 | 82 |
|  |  | -51 | 32 | 11 |  |  |
| **ROI in the right angular gyrus (rostroventral area 39)** | | | | | | |
| L Inferior Frontal Gyrus  L Orbital Gyrus | caudal area 45;  inferior frontal sulcus;  lateral area 12/47 | -54 | 32 | 11 | 0.000 | 211 |
|  |  | -42 | 26 | -10 |  |  |
|  |  | -54 | 23 | 20 |  |  |
| R Inferior Frontal Gyrus  R Middle Frontal Gyrus | dorsal area 44;  caudal area 45;  inferior frontal sulcus;  ventral area 44;  ventral area 9/46 | 57 | 17 | 14 | 0.001 | 171 |
|  |  | 48 | 23 | 29 |  |  |
|  |  | 51 | 29 | 17 |  |  |
| **ROI in the left precuneus (medial area 7)** | | | | | | |
| L Supramarginal gyrus  L Angular Gyrus  L Superior Parietal Lobule | rostrodorsal area 40;  rostroventral area 40;  caudal area 40;  rostrodorsal area 39;  rostral area 7;  postcentral area 7 | -48 | -37 | 38 | 0.000 | 316 |
|  |  | -27 | -58 | 53 |  |  |
|  |  | -57 | -34 | 41 |  |  |
| L Inferior Frontal Gyrus  L Middle Frontal Gyrus | inferior frontal fulcus;  caudal area 45;  ventral area 9/46 | -39 | 35 | 11 | 0.002 | 156 |
|  |  | -42 | 32 | 23 |  |  |
| **ROI in the right precuneus (medial area 7)** | | | | | | |
| L Inferior Frontal Gyrus  L Middle Frontal Gyrus | inferior frontal sulcus;  caudal area 45;  ventral area 9/46 | -42 | 29 | 11 | 0.002 | 152 |
|  |  | -39 | 35 | 26 |  |  |
|  |  | -39 | 17 | 23 |  |  |
| L Supramarginal Gyrus | rostrodorsal area 40 | -45 | -37 | 38 | 0.051 | 68 |
|  |  | -42 | -46 | 62 |  |  |
|  |  | -60 | -28 | 35 |  |  |
| **ROI in the left precuneus (area 31)** | | | | | | |
| L/R Supramarginal Gyrus  L Superior Parietal Lobule  L Postcentral Gyrus | rostrodorsal area 40;  rostroventral area 40;  caudal area 40;  rostral area 7;  caudal area 7;  intraparietal area 7;  area 2 | -57 | -34 | 44 | 0.000 | 795 |
|  |  | -57 | -16 | 29 |  |  |
|  |  | -51 | -37 | 38 |  |  |
| L Inferior Frontal Gyrus  L Middle Frontal Gyrus | inferior frontal sulcus;  caudal area 45;  ventral area 9/46 | -48 | 29 | 11 | 0.002 | 152 |
|  |  | -48 | 38 | 8 |  |  |
|  |  | -39 | 35 | 11 |  |  |
| Cerebellum | lateral area 37 | -39 | -58 | -28 | 0.044 | 72 |
|  |  | -39 | -43 | -19 |  |  |
| **ROI in the right precuneus (area 31)** | | | | | | |
| L Inferior Frontal Gyrus | inferior frontal sulcus;  caudal area 45 | -48 | 29 | 11 | 0.016 | 91 |
|  |  | -39 | 29 | 11 |  |  |
|  |  | -33 | 29 | 23 |  |  |
| L Supramarginal gyrus  L Superior parietal lobule | rostrodorsal area 40;  rostroventral area 40;  caudal area 40;  rostral area 7;  postcentral area 7 | -54 | -28 | 56 | 0.000 | 300 |
|  |  | -57 | -16 | 29 |  |  |
|  |  | -42 | -37 | 47 |  |  |

R/L, right/left hemisphere

**
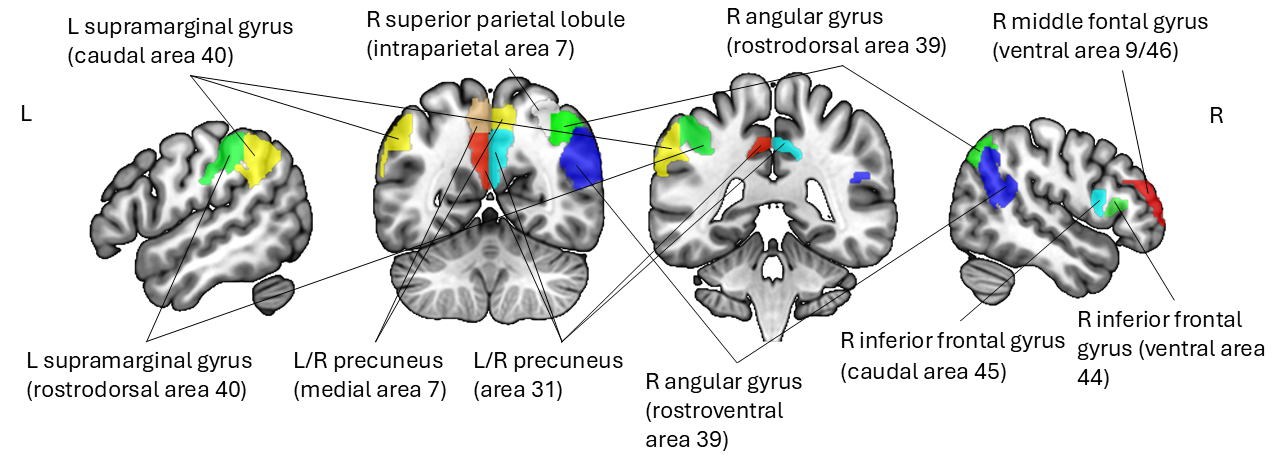
**

**Supplementary Figure 9.** ROIs for the TMFC analysis


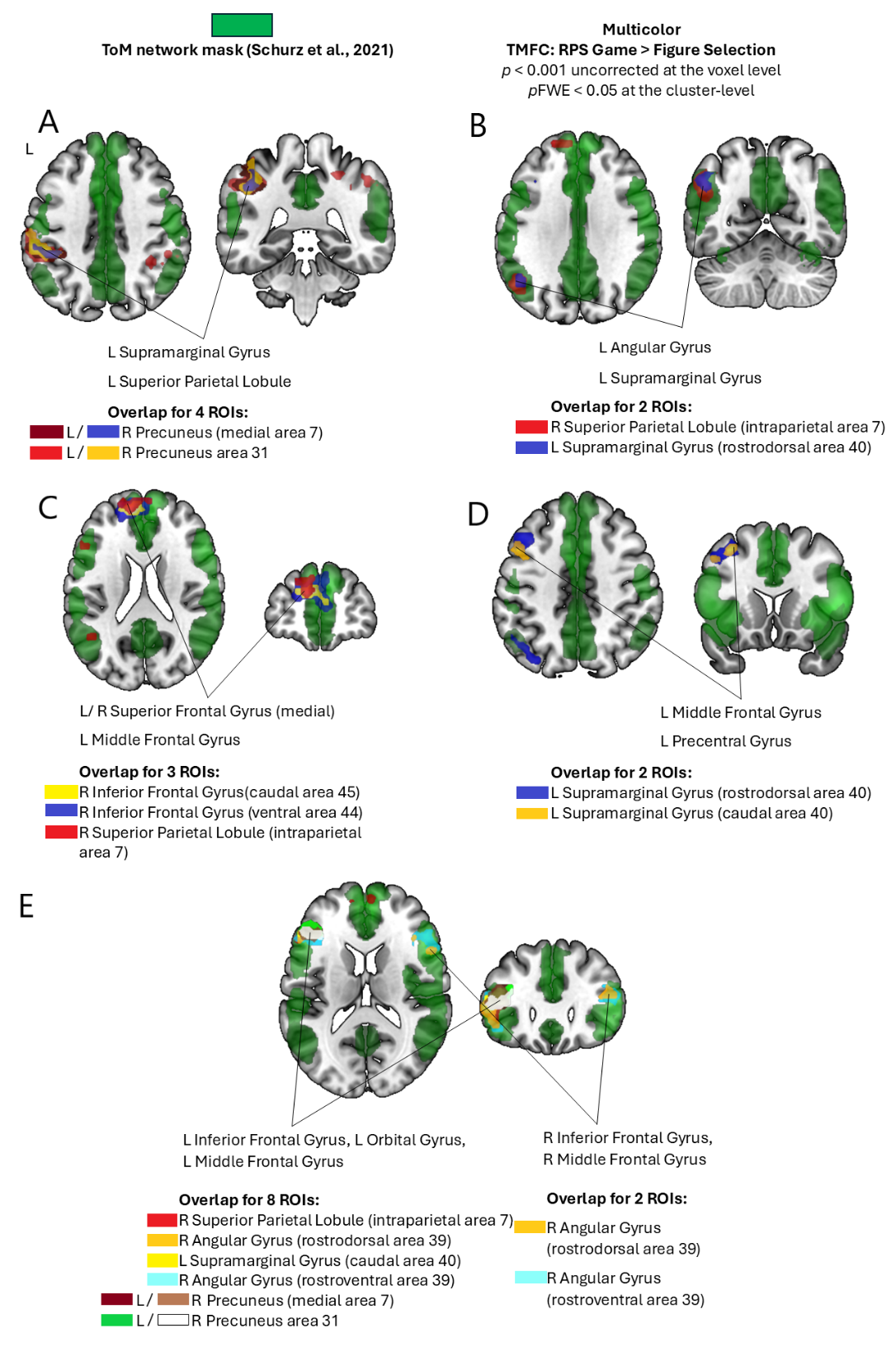


**Supplementary Figure 10.** Overlapping clusters of RPS-induced increases in TMFC across multiple ROIs are shown in relation to their overlap with the ToM network clusters, based on the ToM network masks from the meta-analysis by Schurz et al. (2021) (<https://neurovault.org/collections/9936/>, accessed September 3, 2024). (A) Absence of overlap for the cluster in the left supramarginal gyrus and superior parietal lobule. (B) Overlap for the cluster in the left angular and supramarginal gyri. (C) Overlap for the cluster in the bilateral medial superior frontal gyrus and left middle frontal gyrus. (D) Absence of overlap for the cluster in the left middle frontal and precentral gyri. (E) Overlap for the cluster in the left inferior frontal, orbital, and middle frontal gyri and for the cluster in the right inferior frontal and middle frontal gyri.
